# Uprighting Impacted Mandibular Second Molar Using a Skeletal Anchorage: A Case Report

**DOI:** 10.3390/dj8040129

**Published:** 2020-11-18

**Authors:** Federica Altieri, Rosanna Guarnieri, Martina Mezio, Gabriella Padalino, Angela Cipollone, Ersilia Barbato, Michele Cassetta

**Affiliations:** Department of Oral and Maxillofacial Sciences, “Sapienza” University of Rome, 6-00161 Rome, Italy; federica.altieri@uniroma1.it (F.A.); rosanna.guarnieri@uniroma1.it (R.G.); martinamezio@gmail.com (M.M.); gabriellapadalino@gmail.com (G.P.); angela.cipollone@outlook.it (A.C.); ersilia.barbato@uniroma1.it (E.B.)

**Keywords:** molar impaction, mandibular second molar, molar uprighting, impacted teeth, orthodontics, TADs, miniscrew

## Abstract

The aim of this case report is to present an innovative combined orthodontic-surgical technique to disimpact mandibular second molar (MM2) using an orthodontic miniscrew and an elastic chain. The impact on the Oral health-related quality of life (OHRQoL) was also evaluated. Using the present techinique, it is possible to expose the impacted tooth, insert a self-drilling miniscrew in the retromolar area, and remove the bud of third mandibular molar. At the same time the orthodontic force is applied with the use of an elastomeric chain that connects the head of miniscrew and vestibular and oral buttons bonded on MM2. A close traction is performed for the whole treatment time without the reactivation of the elastic force. The use of skeletal anchorage allowed the disimpaction of impacted MM2 in a short treatment time (about three months) avoiding the typical biomechanical side effects of traditional orthodontic appliance and increasing the effectiveness of the treatment. Further studies are necessary to evaluate the real advantages and disadvantages of this combined orthodontic-surgical approach.

## 1. Introduction

Mandibular second molar (MM2) impaction is an infrequent event [1] compared to other dental anomalies [2,3,4], characterized by an increase in prevalence over the years [5,6]. A high impaction risk of MM2 is associated with a mesial angulation form, which is also the most common (88%) [1,5,6]. When the initial mesial inclination of MM2 is greater than 20°/30° this is significantly associated with MM2 impaction [5,6].

Management of mesially-angulated impacted MM2 often requires an interdisciplinary approach. The treatment options depend both on the degree of tooth inclination and the required tooth movement. The position of a slightly-tipped MM2 can be corrected by placing a brass wire separator between the teeth [7,8,9]. A more severe inclination needs a combined orthodontic-surgical technique [10,11,12]. The best time to treat impacted MM2s is when the development of the MM2 roots is still incomplete [12]. The best surgical procedure and the total treatment time for the MM2 disimpaction require an accurate assessment of the initial position of the impacted second molar. Nowadays, with the introduction of miniscrews, it has become possible to solve the MM2 impaction in a short treatment time, reducing and avoiding biomechanical side effects and increasing the effectiveness of the treatment [13]. The temporary anchorage devices (TADs) have the advantages of being relatively inexpensive, easy to place and remove. Miniscrews can be inserted in many places in the maxilla and mandible [14,15,16] and are predictable enough to be used routinely in dental practice. In addition, a minimal degree of cooperation is required during the treatment. The aims of this case report are:

- to show an innovative combined orthodontic-surgical procedure to solve a moderately mesially-impacted mandibular second molar (MM2) using an orthodontic miniscrew with an elastic chain;
- to evaluate the impact of this procedure on the Oral health–related quality of life (OHRQoL).

## 2. Materials and Methods

### 2.1. Case Presentation

The present procedure, used at the Department of Oral and Maxillo-Facial Sciences of “Sapienza” University of Rome, allows MM2 disimpaction employing a submucosal orthodontic miniscrew with an elastic chain. A 12-year-old Caucasian female patient was referred to the Department of Orthodontics of “Sapienza” University of Rome to solve several tooth impactions. The patient’s medical history showed nothing remarkable. The patient referred to a previous orthodontic treatment with a lingual arch in the mandible to gain space. Intraoral examination revealed a molar Class II malocclusion with not centered midlines, an overjet of 2 mm and an overbite of 5 mm. The patient showed a severe crowding of maxilla (7 mm) with no space for maxillary canines eruption. A moderate crowding was present in the mandible (6 mm). Temporomandibular joints were healthy.

**Figure 1:**
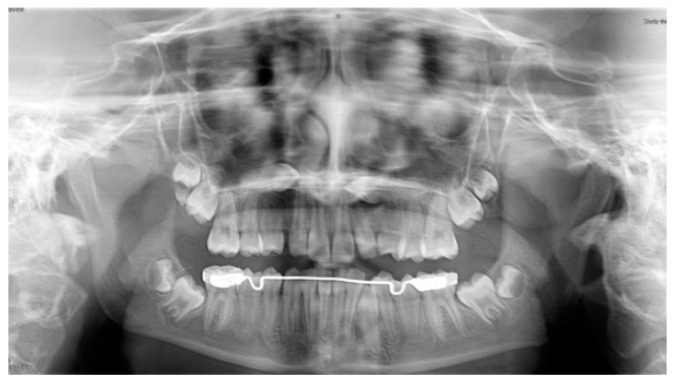
Pretreatment panoramic radiograph showing bilaterally impacted MM2 and upper canines.

The panoramic radiograph showed the presence of several impacted teeth (Figure 1). Angle of inclination of MM2 was measured, as described by Evans [17]. The angle of the left MM2 was 30°, the angle of the right MM2 was 40°. Cephalometric measurements were performed using lateral cephalometric radiography that showed a Class I skeletal relationship (ANB = 2°), brachyfacial growth pattern (MP-SN = 30.5°; FMA = 17.5°) and palatal inclination of the upper incisors (U1—ANS-PNS = 107.0°).

### 2.2. Treatment Plan

The objectives of treatment were to solve:

- maxillary canines impaction (1.3 and 2.3);
- MM2 impaction (3.7 and 4.7)

The following three treatment options to solve MM2 impaction were presented: a conventional orthodontic treatment with lingual arch with a posterior arm; a cantilever arm supported by a miniscrew or teeth; a close traction with a retromolar miniscrew connected to an elastic chain.

The patient’s need was to solve the dental impactions in the shortest possible time without masticatory limitation. The treatment plan was to remove the impacted maxillary canines and to solve MM2 impaction using a combined orthodontic-surgical technique with orthodontic miniscrews and an elastic chain. The patient was informed of the risks, advantages and disadvantages of this therapeutic approach and provided written informed consent to undergo the procedures.

The advantages of this technique are: the absence of orthodontic device in the oral cavity (submerged technique) allowing the maintenance of good oral hygiene without masticatory limitation. Furthermore, the orthodontic traction was not reactivated, reducing the number of appointments and improving the patient’s comfort.

The main disadvantage was the need to perform a flap to remove the miniscrew.

The guidelines of the declaration of Helsinki were followed. The investigation was independently reviewed and approved by the local ethics committee of Policlinico Umberto I-Sapienza University of Rome (Rif. 5951 on date 22 April 2020).

### 2.3. Surgical-Orthodontic Treatment of an Impacted MM2

The day of surgery the patient was treated by a single operator, an expert orthodontist and oral surgeon (MC). Local anesthesia with mepivacaine (20 mg/mL) associated with epinephrine in the ratio 1:100,000 (Optocain, Molteni Dental S.r.l) was administered. A mucoperiosteal flap extending from the first molar to the retromolar area both on the buccal and lingual side was reflected to expose the bone surrounding the impacted MM2 (3.7) and the third mandibular molar (MM3) bud. Bone was carefully removed, as required, for the MM3 germectomy. Immediately afterwards, a self-drilling miniscrew was inserted in the retromolar area by a cordless screwdriver with a torque calibration system (ISD900, NSK, Tochigi, Japan). The fixture insertion torque was set at 40 N/cm.

**Figure 2:**
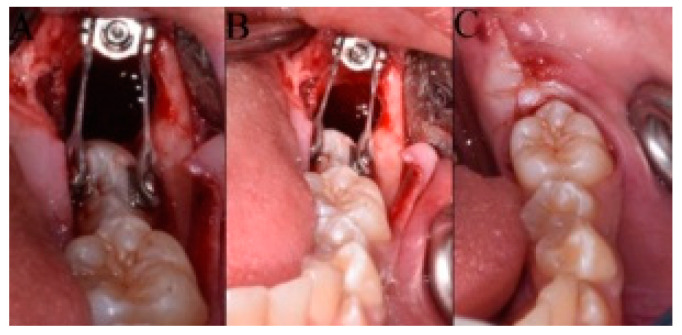
(**A**,**B**) View of inserted self-drilling miniscrew in the retromolar area, the buccal and oral metal brackets on the crown of mandibular second molar (MM2) and the orthodontic traction with the elastomeric chain; (**C**) Surgical incision closure with sutures.

The site and angle of insertion, the length and the diameter of titanium miniscrew (BENEfit, psm, Tuttlingen, Germany) were determined in the planning phase on a panoramic radiograph (length 13 mm; 2.3 mm diameter) and clinically confirmed during the surgery. After the self-drilling miniscrew insertion, two metal brackets were bonded on the buccal and oral surfaces of MM2 crown. At the end of surgery an elastic chain was secured to the miniscrew head and connected to the metal brackets bonded on buccal and oral surfaces of MM2 crown (Figure 2A,B); a close/submerged traction was performed. On the head of the miniscrew was screwed an abutment with two brackets (Figure 2A,B); an elastic chain was applied to each bracket (Figure 2B). Each elastic chain was activated between the bonded button on the surface of the second molar to the bracket of the miniscrew. A short elastic chain was used that was stretched approximately twice. Finally, the surgical site was sutured (Figure 2C).

Oral health–related quality of life (OHRQoL) was assessed using the Italian version of the short-form oral health impact profile with 14 questions (OHIP-14) that represent 7 dimensions of OHRQOL: functional limitation, physical pain, psychological discomfort, physical disability, psychological disability, social disability, and handicap [18,19]. The patients received the questionnaire after being instructed in its use. The self-administered questionnaire was filled out by the patients preoperatively (baseline; T0), 3 days post-surgery (T1), and 7 days post-surgery (T2). Answers were made on an ordinal, 5-point, adjectival scale (0 never, 1 hardly ever, 2 occasionally, 3 fairly often, 4 very often). OHRQoL is characterized by summary scores of the OHIP-14 items. Higher scores reveal a stronger negative influence on OHRQoL.

Ibuprofen was recommended for pain control. Antibiotic therapy (1 gr of amoxicillin) was prescribed 1 h before intervention and twice a day for 5 days.

The patient was checked every month until the uprighting of MM2 was complete.

The same elastic chain was not changed for the whole time of therapy.

**Figure 3:**
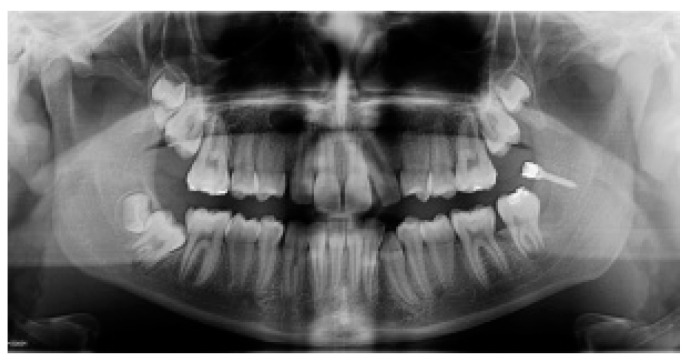
Post-treatment panoramic radiograph showing the correct inclination of 3.7 before miniscrew removal.

The MM2 was considered upright when the mesial marginal ridge was above the distal contours of first mandibular molar (MM1) (Figure 3).

**Figure 4:**
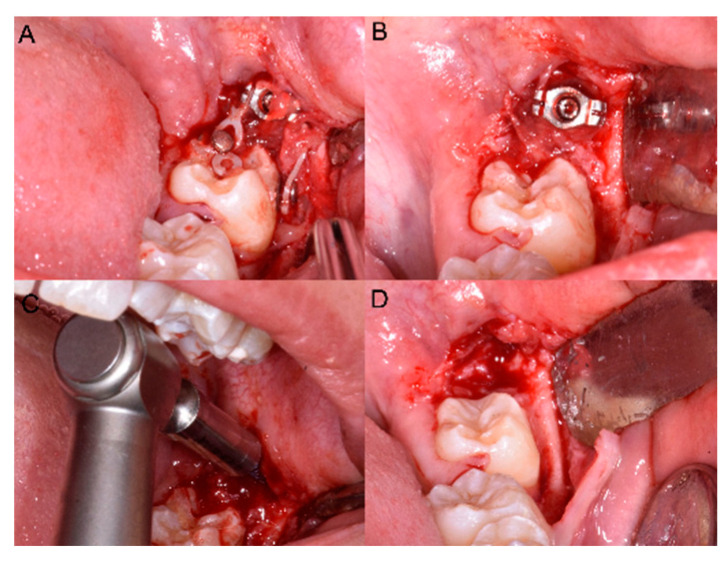
(**A**,**B**) The elastic chain, brackets and (**C**) the miniscrew removal. (**D**) The final position in the arch of 3.7.

At the end of therapy the miniscrew was removed (Figure 4) and the other impacted MM2 was treated both with the same miniscrew and with the same orthodontic surgical procedure.

## 3. Results

No important adverse events or side effects were recorded.

The OHIP-14 filled out by the patient before the treatment (T0), and 3 (T1) and 7 (T2) days after surgery had a total score of 15 points (T0 = 3; T1 = 8; T2 = 4). A deterioration in OHRQoL was observed only after 3 days of surgery; an improvement in OHRQoL was observed after 7 days of the procedure with almost a complete restoration (OHIP-14 = 4) of pre-treatment value.

The MM2 impaction was corrected in 92 days.

## 4. Discussion

Management of MM2 impaction is considered very difficult and unpredictable and is a challenge for both orthodontist and oral surgeon. Close collaboration between the two specialists is needed to achieve the MM2 disimpaction. An early diagnosis and early treatment are the keys for successful correction of MM2 impaction. In addition, it is important to disimpact the MM2 as soon as it is diagnosed because of the contact with the MM1 that could cause root resorption, caries, and periodontal problems.

Treatment options for an impacted MM2 can include extraction, orthodontic uprighting, surgical uprighting, and surgical-orthodontic approach [20,21,22]. Many orthodontic appliances and techniques have been suggested for the uprighting of moderately mesially-impacted molars [20,21,22]. Nowdays, thanks to miniscrews, it is possible to reduce biomechanical side effects and to solve the MM2 impaction in a short treatment time [23,24,25].

The present technique allows, in a single appointment, the exposure of the impacted tooth, the insertion of the screw, the extraction of the third molar bud and the activation of the orthodontic traction.

Regarding the combined use of miniscrews and elastic chains, few similar studies exist in the literature [24,25]. Miyahira et al. [24] treated an impacted MM2 with a miniplate and an elastic chain. Although the technique appears to be predictable and quick, the use of two miniscrews, and the difficulty of maintaining oral hygiene around the miniplate, could cause a grater patient discomfort.

Zen et al. [25] described the uprighting of a MM2 using a miniscrew with a Nichel-Titanium closed coil spring, from the miniscrew to a single lingual button, replaced every month.

Unlike the techniques described above, the present procedure is a close traction technique. A miniscrew is inserted in the retromolar area and two brackets or buttons attachments are bonded on the crown of MM2; an elastic chain is connected from the head of the miniscrew to the two buttons, minimizing the size of the orthodontic device, reducing chair time and improving the patient comfort compared to complex segmental biomechanics. The force application generates a counterclockwise moment, allowing control of the movement and the collateral effects and, consequently, promoting rapid disimpaction and distalization of the crown.

Moreover, this procedure allows an absolute control of the anchorage and no unwanted movement of adjacent teeth [23,24,25].

The retromolar area seems particularly suitable for screw insertion, considering the presence of compact cortical bone tissue which immediately provides excellent primary stability. According to Finotti et al., after third molar extraction, the creation of a cortico-medullar void distal to the second molar, or appositely surgically performed is important to reduce the treatment time [26].

Nowadays, computer-guided miniscrew-supported orthodontic appliances can be realized [27,28] thanks to dedicated software that matches the stereolithography STL files of the dental arch with the DICOM images of the CBCT. However, in the present technique, the miniscrew has been inserted freehand because in the retromolar area there is usually a sufficient quality and quantity of bone. The insertion of a miniscrew in this area does not require a CT scan but the information provided by an orthopanoramic radiograph is sufficient. In addition, the simplicity of the orthodontic appliance does not require a custom-made appliance.

## 5. Conclusions

In conclusion, it can be stated that the combined use of miniscrew and elastic chains could be a valid procedure for moderate mesially-impacted MM2. In a single surgical time the MM3 germectomy, the miniscrew insertion and the orthodontic traction are performed, without a negative impact on the OHRQoL. Certainly, an early diagnosis can prevent impaction of these teeth, enabling a more conservative treatment approach, so a radiographic follow-up of the dental eruption process should be encouraged to observe an early delay in the eruption of the MM2. It must be highlighted that the efficacy of this approach must be further investigated to evaluate the real advantages and disadvantages of this technique.
